# Enamel biomineralization under the effects of indomethacin and celecoxib non-steroidal anti-inflammatory drugs

**DOI:** 10.1038/s41598-022-19583-w

**Published:** 2022-09-22

**Authors:** Juliana de Lima Gonçalves, Ana Caroline Alves Duarte, Luciano Aparecido Almeida-Junior, Fabrício Kitazono de Carvalho, Alexandra Mussolino de Queiroz, Maya Fernanda Manfrin Arnez, Lúcia Helena Faccioli, Francisco Wanderley Garcia Paula-Silva

**Affiliations:** 1 Departament of Pediatric Clinics, School of Dentistry of Ribeirão Preto at University of São Paulo, Avenida do Café, S/N., Ribeirão Preto, SP 14040-904 Brazil; 2 Departamento de Análises Clínicas, Toxicológicas e Bromatológicas, Faculdade de Ciências Farmacêuticas de Ribeirão Preto, Universidade de São Paulo, Avenida do Café, S/N, Ribeirão Preto, São Paulo 14040-903 Brazil

**Keywords:** Paediatric dentistry, Special care dentistry

## Abstract

The aim of this study was to explore the effects of nonsteroidal anti-inflammatory drugs on biomineralization of enamel. Sixty C57Bl6 male mice were used, which were assigned into three groups: celecoxib (n = 20) or indomethacin (n = 20) treatment for a period of 28 days or received no medication (control group, n = 20). Visual inspection and microcomputed tomography were used to analyze enamel morphology. Scanning electron microscopy–Energy dispersive X-ray and Knoop microhardness test were used to quantify chemical element content (Ca, P, C, O) and enamel microhardness, respectively. Tissues were collected to investigate the synthesis, activity or nuclear translocation of metalloproteinase-20, transcription factor *Runx2*, dentin sialoprotein and cyclooxygenase-2 enzyme by means of immunohistochemistry, in situ zymography and indirect immunofluorescence. Treatment with indomethacin and celecoxib reduced the Ca and P content, microhardness and mineral density in enamel. Treatment with nonsteroidal anti-inflammatory drugs caused an accumulation of metalloproteinase-20 and overall increased enzymatic activity in enamel matrix, while the synthesis of the transcription factor Runx2 was inhibited by these drugs. Interestingly, indomethacin inhibited Runx2 translocation to the nucleus whereas celecoxib did not. Those findings show that non-steroidal anti-inflammatory drugs impact the enamel biomineralization and could be involved in the etiology tooth enamel defects if used during the period of tooth formation and mineralization.

## Introduction

Dental enamel defects (DED) can be classified as qualitative and quantitative. Qualitative defects are those characterized by hypomineralization of the enamel where it remains at normal thickness, but with defective enamel mineralization, observed by diffuse or demarcated white to yellow–brown opacities. Quantitative defects, on the other hand, are known as enamel hypoplasia, which is marked by a reduction in enamel thickness^1^. The etiology of DED is multifactorial^2^ and it is widely accepted that systemic, hereditary and environmental factors might be involved^3,4^.

Amelogenesis is the process by which dental enamel, a hard structure composed of an inorganic mineralized matrix (approximately 95 wt%), is formed^5,6^. Amelogenesis can be classified into the following phases: morphogenetic, differentiation, secretion, maturation and protection^7^. It is hypothesized that DED occur during both the secretion phase of amelogenesis, a stage in which proteins such as amelogenin are deposited in the matrix with the role of guiding the formation of crystals, and also in the maturation phase, in which the organic content is removed through the action of enzymes such as metalloproteinase-20 (MMP-20) where biomineral content deposition occurs^4^. Amelogenesis is a complex process which includes the presence of components that plays important functions so the dental enamel can form properly^8^.

MMP-20 is an enzyme that is present in the extracellular matrix of enamel and found in the early stages of matrix secretion^9^. Mutations in this enzyme are related to the occurrence of *amelogenesis imperfecta*^10^. Its main known function is to carry out the degradation of matrix proteins, leading to a replacement of the space occupied by the proteins by a biomineral content^9^. Runx2 transcription factor, on the other hand, is known for its participation in bone development, and mutations in this factor are related to cleidocranial dysplasia, a condition characterized by delayed junction of the cranial fontanelles, hypoplasia of the clavicle, and dental anomalies such as supernumerary teeth and delays in tooth eruption^11^. Regarding tooth development, Runx2 would be associated with regulating the accumulation of enamel matrix proteins and the synthesis of KLK4, an enzyme present in the stages of dental secretion and mineralization, which has the main function of degrading matrix enzymes^10,12^ DSPP (dentin sialophosphoprotein) is a protein synthesized during dentin formation that plays an important role in dentin formation. DSPP deficiency or mutation is related to the occurrence of type III *dentinogenesis imperfecta*, a condition that can lead to reduced dentin thickness and increased pulp chamber^13^. DSPP is a large protein that is cleaved and gives rise to DSP (dentin sialoprotein) and DPP (dentin phosphoprotein)^14^.

In addition, it is known that endogenous and exogenous factors that impair the amelogenesis stages potentially could lead to the occurrence of DED^3,4^. The effects of the use of medication during the development of dental enamel which occur in the first 3 years of life is widely studied^2,15^. NSAIDs are a class of drugs commonly prescribed for children, and they are used for the treatment of inflammatory process^15,16^. The anti-inflammatory effect of NSAIDs is due to cyclooxygenase (COX) inhibition, which can be selective or non-selective. Non-selective inhibitors such as indomethacin act by inhibiting both COX-1 and COX-2^17^. Otherwise, celecoxib is a selective inhibitor, causing the inhibition of COX-2^18–21^.

Knowing the function and possible participation of these proteins and enzymes in the tooth biomineralization process, the objective was to identify a possible relationship between non-steroidal anti-inflammatory drugs (NSAIDs) and DED. This study sought to explore the cellular effects caused by the use of nonsteroidal anti-inflammatory drugs during the biomineralization of enamel aiming to understand the mechanisms involved in the occurrence of dental enamel defects.

## Results

### Indomethacin and celecoxib treatment impact enamel composition and microhardness

**Figure 1:**
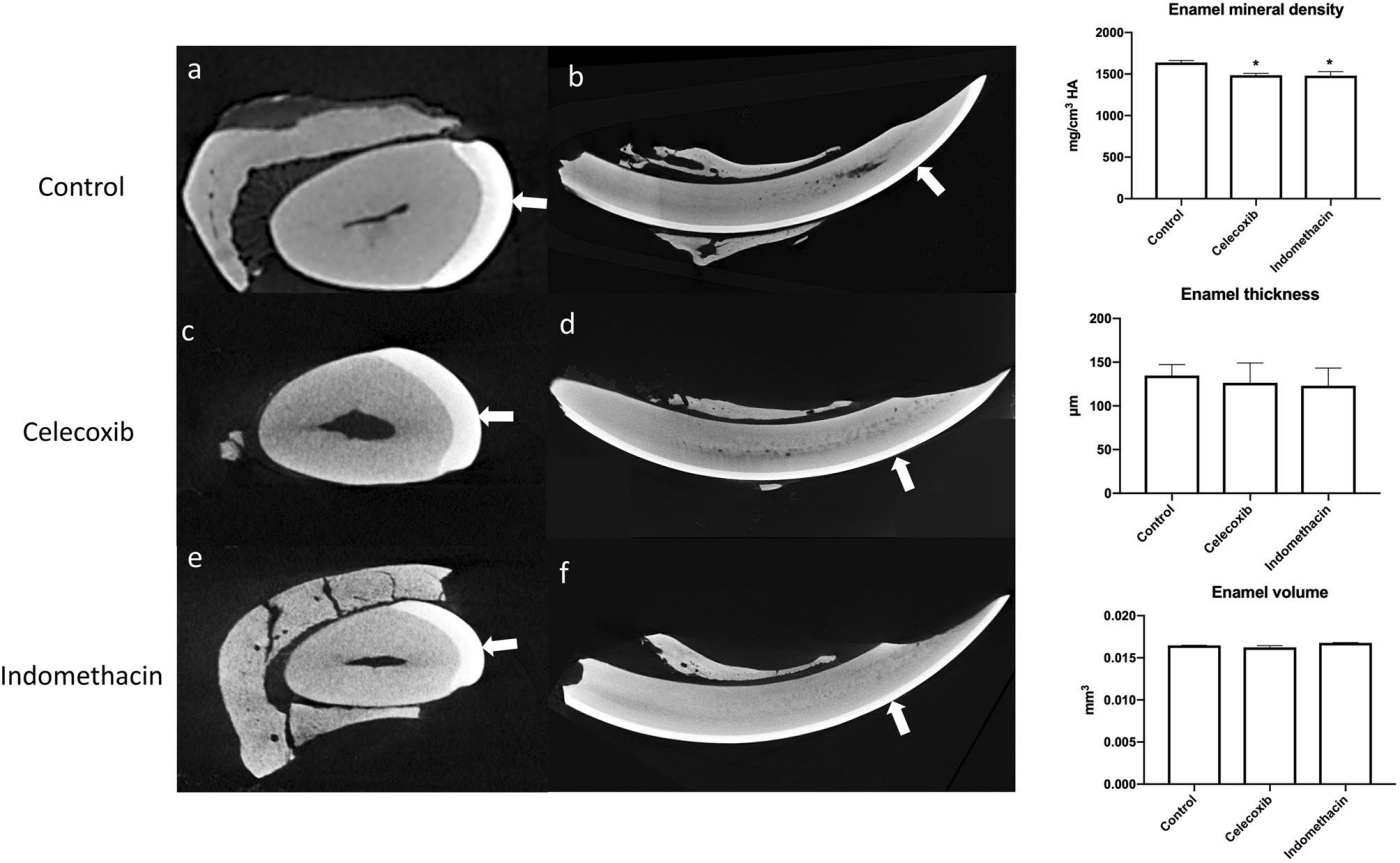
Micro-computed analysis (μCT) of mandibular incisors. Axial images (**a**,**c**,**e**) show the enamel layer of the three groups. In sagittal images (**b**,**d**,**f**) with white arrows that indicates the mature enamel segment which is about to erupt in oral cavity .

**Figure 2:**
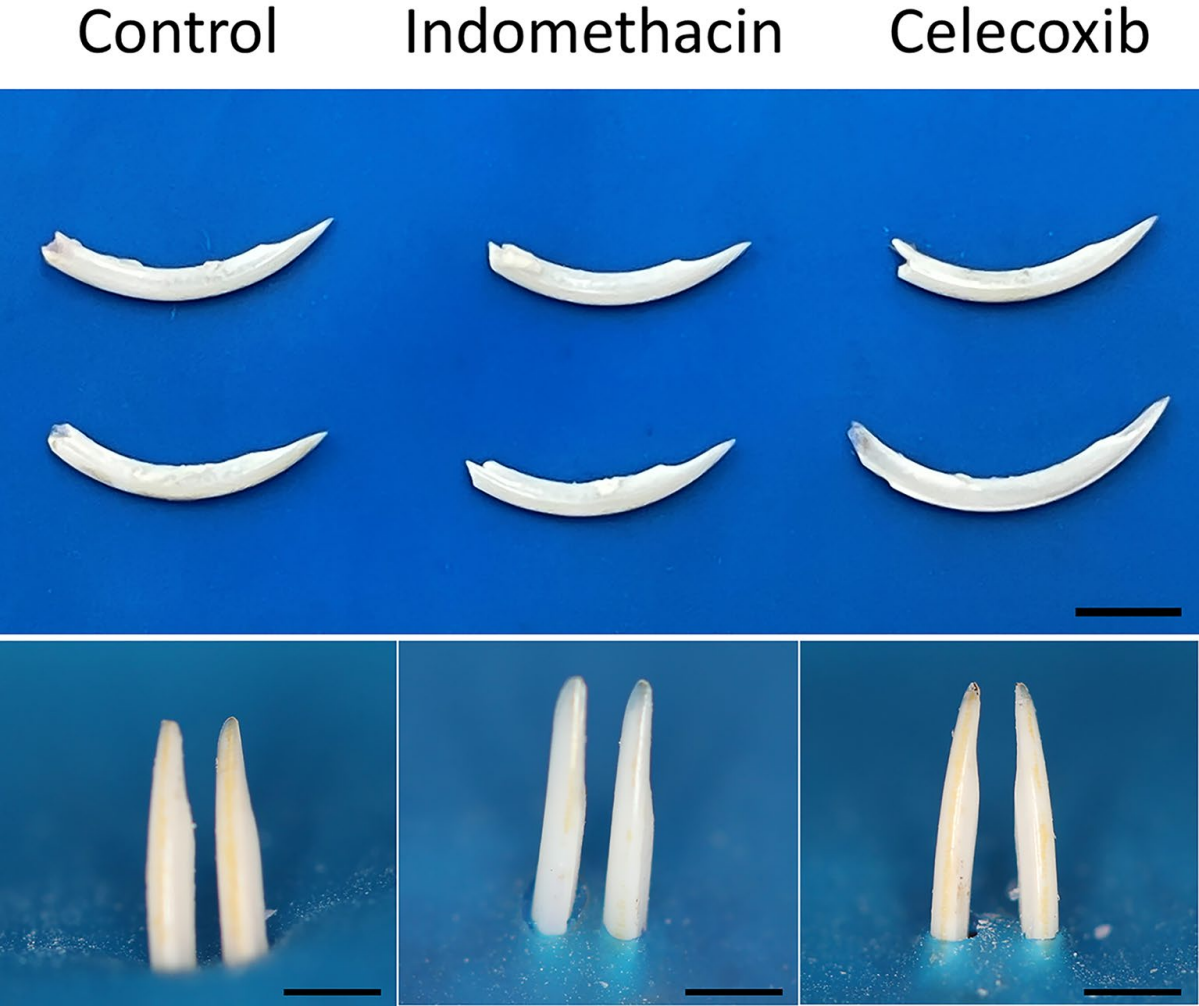
Visual inspection of the dehydrated lower incisors of the mice treated or not with indomethacin and celecoxib (scale = 10 mm).

Animals treated with indomethacin and celecoxib show a significant decrease in the content of calcium and phosphorus when compared to control group. Treatment also increased carbon, but did not change oxygen content compared to control group. Microhardness test was performed and both treatments reduced the enamel hardness compared to the control group (Table 1). Regarding to enamel morphology, treatment did not impact enamel thickness and volume, but significantly reduced the mineral density (Fig. 1). Visual inspection of the enamel, showed no significant structural or color change (Fig. 2).

**Table 1:** Chemical elements content (%/w) and enamel Knoop microhardness (KHN) in lower incisors of mice treated or not with NSAIDs.

Treatment	Ca	P	O	C	Microhardness
Control	46.5 (± 2.1)^A^	22.6 (± 2.3)^A^	17.1 (± 1.9)^A^	13.4 (± 2.6)^A^	200.1 (± 49.9)^A^
Celecoxib	27.1 (± 4.5)^B^	13.2 (± 3.3)^B^	21.1 (± 1.4)^A^	38.3 (± 1.3)^B^	69.47 (± 48.03)^B^
Indomethacin	27.8 (± 1.7)^B^	12.2 (± 1.1)^B^	14.3 (± 1.9)^A^	42.2 (± 6.2)^B^	70.22 (± 47.05)^B^

*Ca* calcium, *P* phosphorous, *O* oxygen, *C* carbon.

### Treatment with NSAIDs impact biomineralization signaling

**Figure 3:**
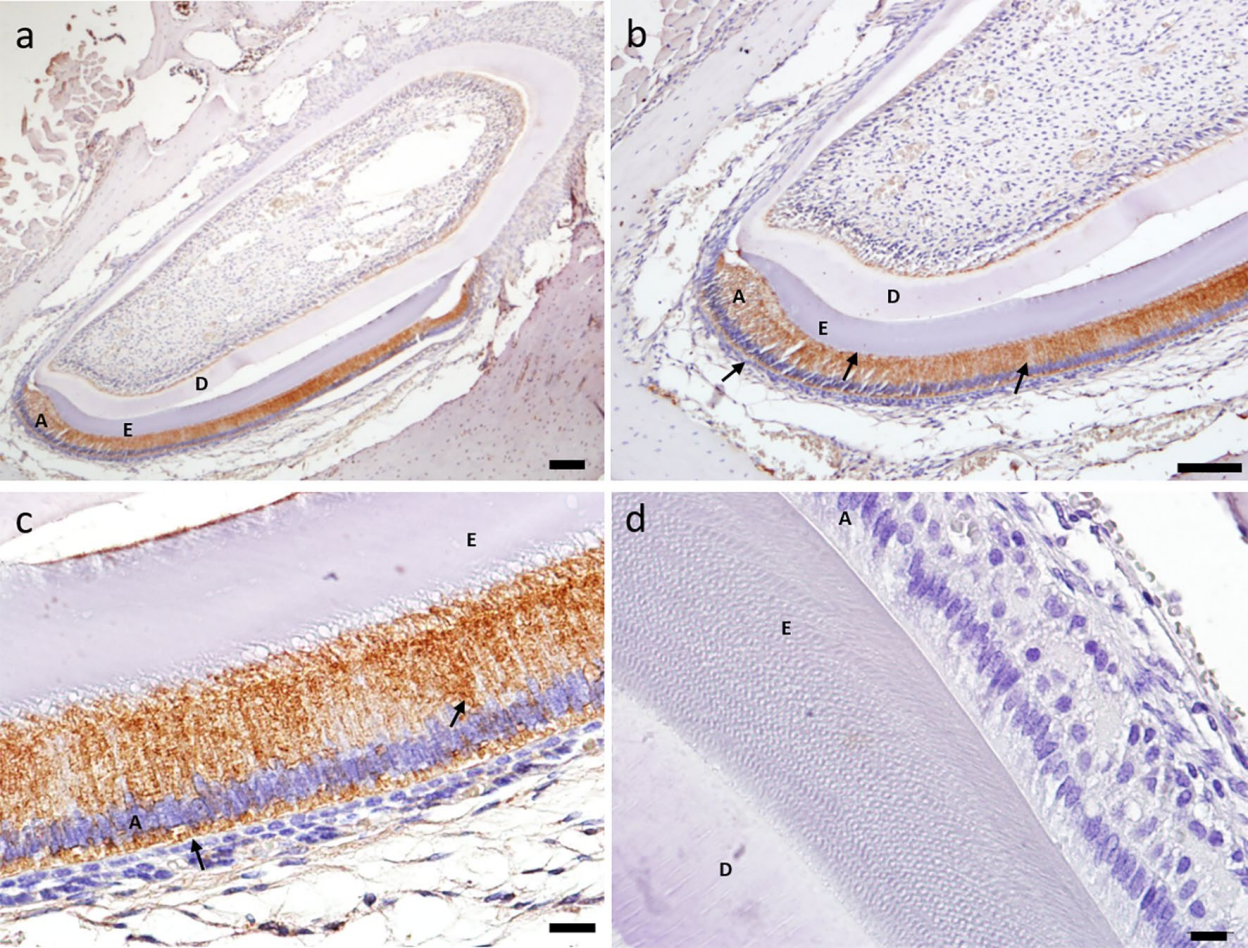
Control group (n = 20) shows an intense COX-2 expression in the ameloblast layer (4×/scale = 200 µm) (**a**), (10×*/*scale = 100 µm) (**b**), (20×/scale = 50 µm) (**c**). In the negative control group, in which the primary antibody was omitted during immunohistochemistry, it was possible to observe no expression of COX-2 (40×/scale = 20 µm) (**d**). A: ameloblasts, E: enamel layer, D: dentin.

COX-2 enzyme was expressed in the control group throughout the entire ameloblastic layer, and its expression decreased as the ameloblasts were more differentiated, forming a gradient line along the layer (Fig. 3).

### Treatment with NSAIDs impact biomineralization signaling

**Figure 4:**
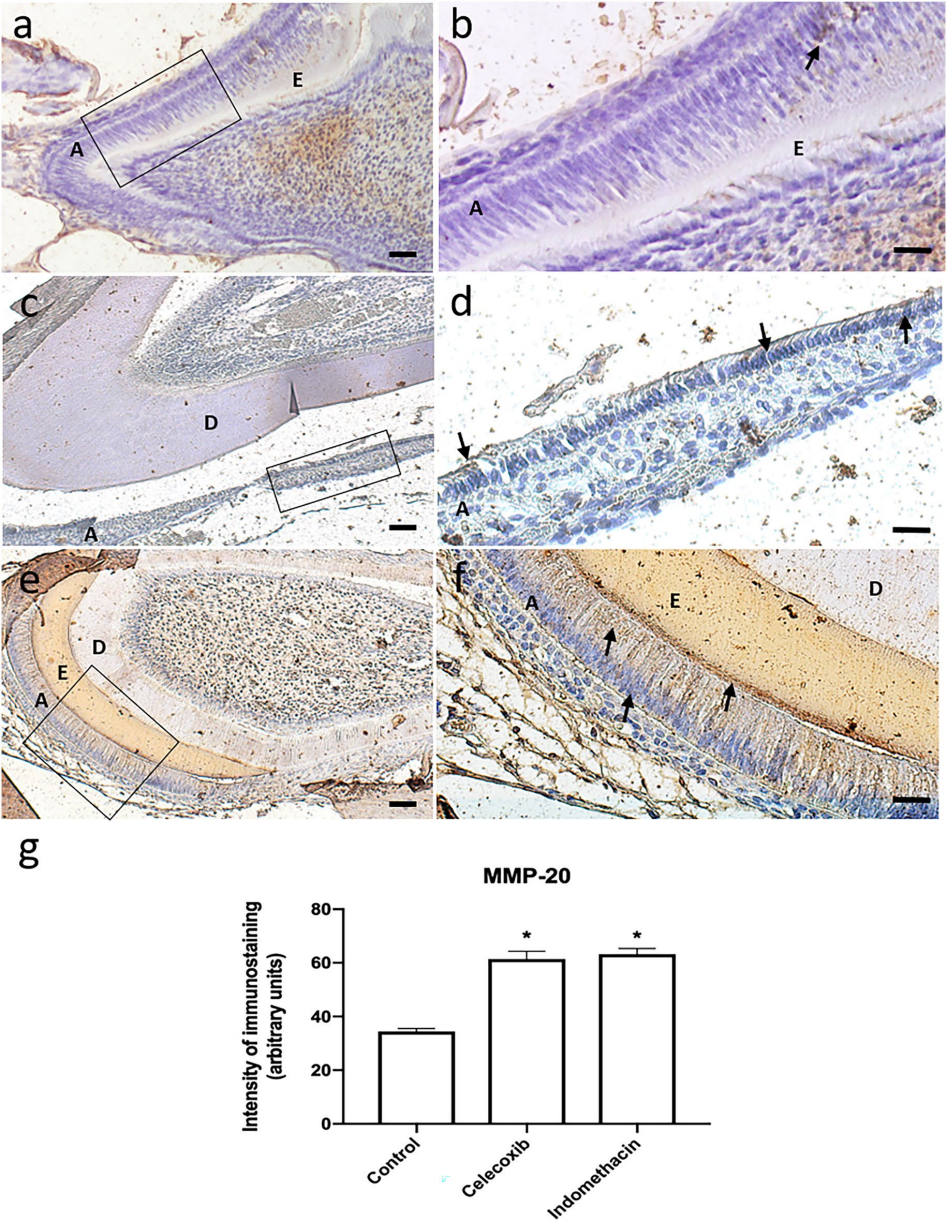
Expression of MMP-20 in the control group (n = 20) (10 × / scale = 100 µm) (**a**), (20×/scale = 50 µm) (**b**). High expression of MMP-20 in the group treated with Celecoxib (n = 20) (10×/scale = 100 µm) (**c**), (20×/scale = 50 µm) (**d**) and Indomethacin (n = 20) (10×/scale = 100 µm) (**e**), (20×/scale = 50 µm) (**f**). Graphical representation of immunostaining quantification of MMP-20 protein synthesis using image deconvolution- median and interquartile range (**g**). A: ameloblasts, E: enamel layer, D: dentin layer.

**Figure 5:**
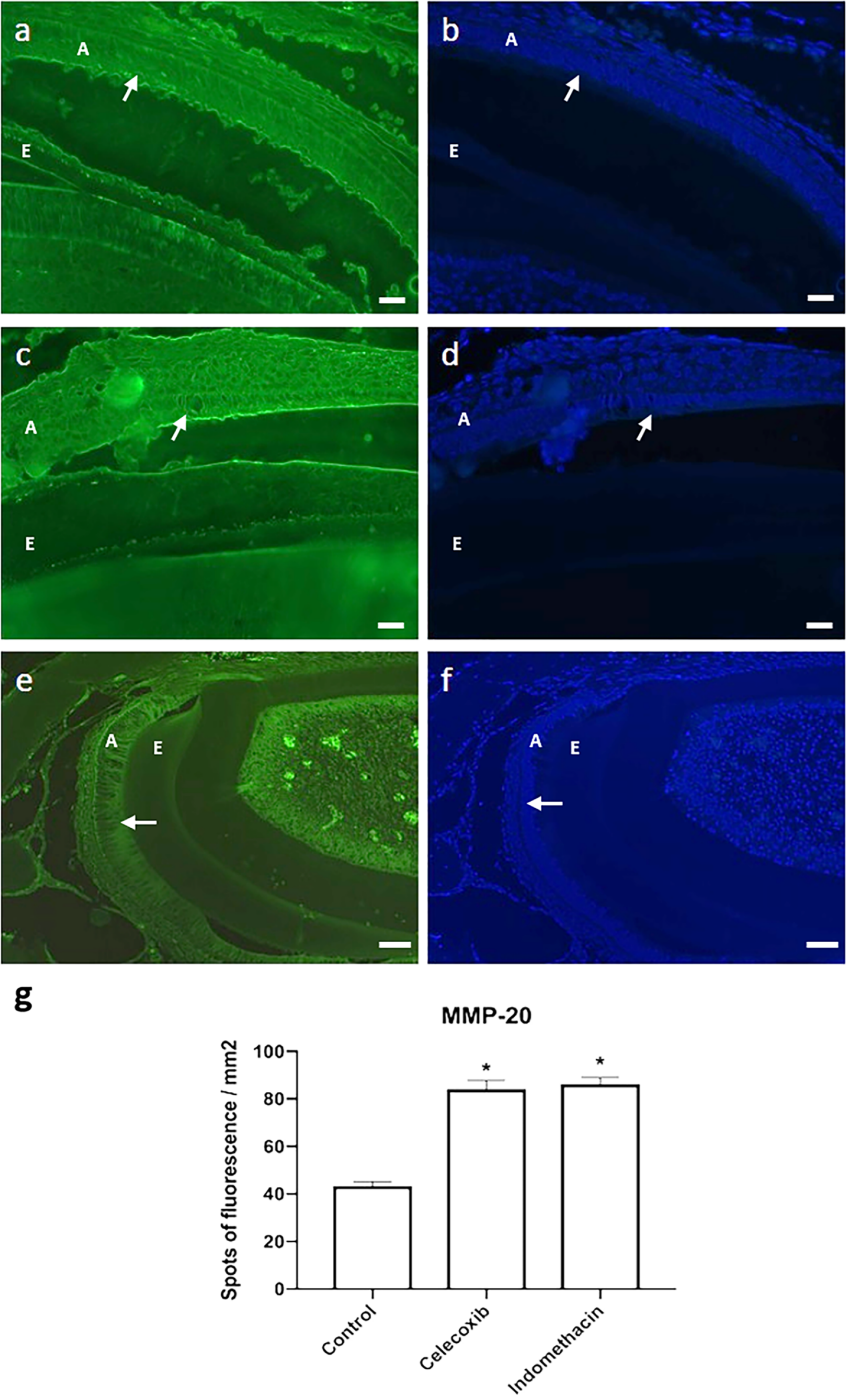
In situ zymography of the control group shows low enzymatic activity (40×*/*scale = 20 µm) (**a**). Celecoxib (40×*/*scale = 20 µm) (**c**) and indomethacin treatment (20×/scale = 50 µm) (**e**) shows an increased synthesis of the enzyme. Depicts nuclear DAPI labeling (40×/scale = 20 µm) (**b** and **d**), (20×/scale = 50 µm) (**f**). A: ameloblasts, E: enamel layer.

MMP-20 synthesis and activity in the enamel layer were induced by indomethacin and celecoxib . In the control group, a lower synthesis of the enzyme was found in the ameloblastic layer and in the non-mineralized enamel matrix (Figs. 4 and 5).

**Figure 6:**
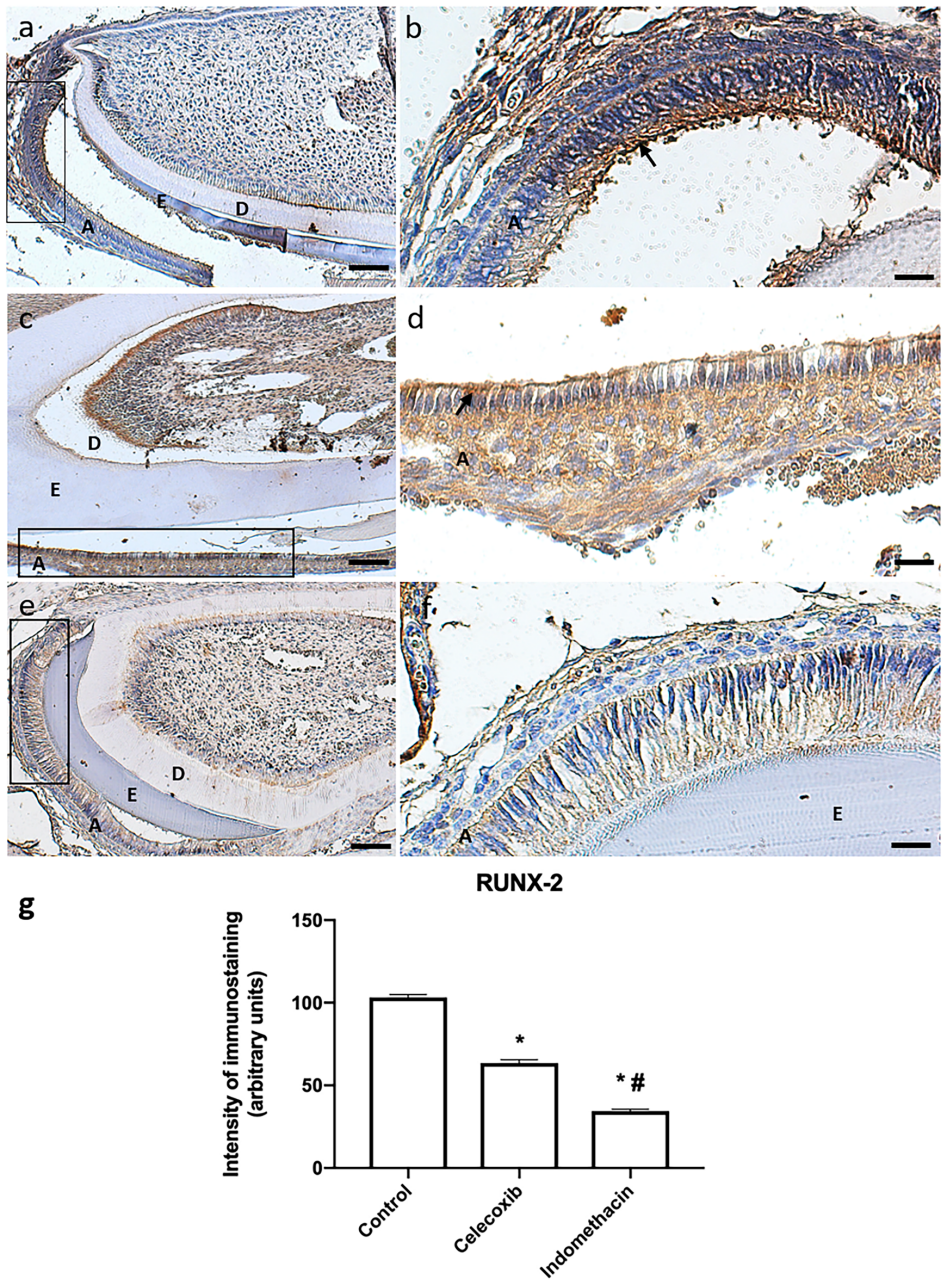
High expression of Runx2 in the control group (n = 20) (10 × */* scale = 100 µm) (**a**), (20×*/*scale = 50 µm) (**b**). Animals treated with Celecoxib (n = 20) presented a slight inhibition of Runx2 in the ameloblast layer (10×*/*scale = 100 µm) (**c**), (20×*/*scale = 50 µm) (**d**). In indomethacin-treated group (n = 10), Runx2 inhibition was even more evident (10×*/*scale = 100 µm) (**e**), (20×*/* scale = 50 µm) (**f**). Graphical representation of immunostaining quantification of Runx2 protein synthesis using image deconvolution- median and interquartile range (**g**). A: ameloblasts, E: enamel layer, D: dentin layer.

**Figure 7:**
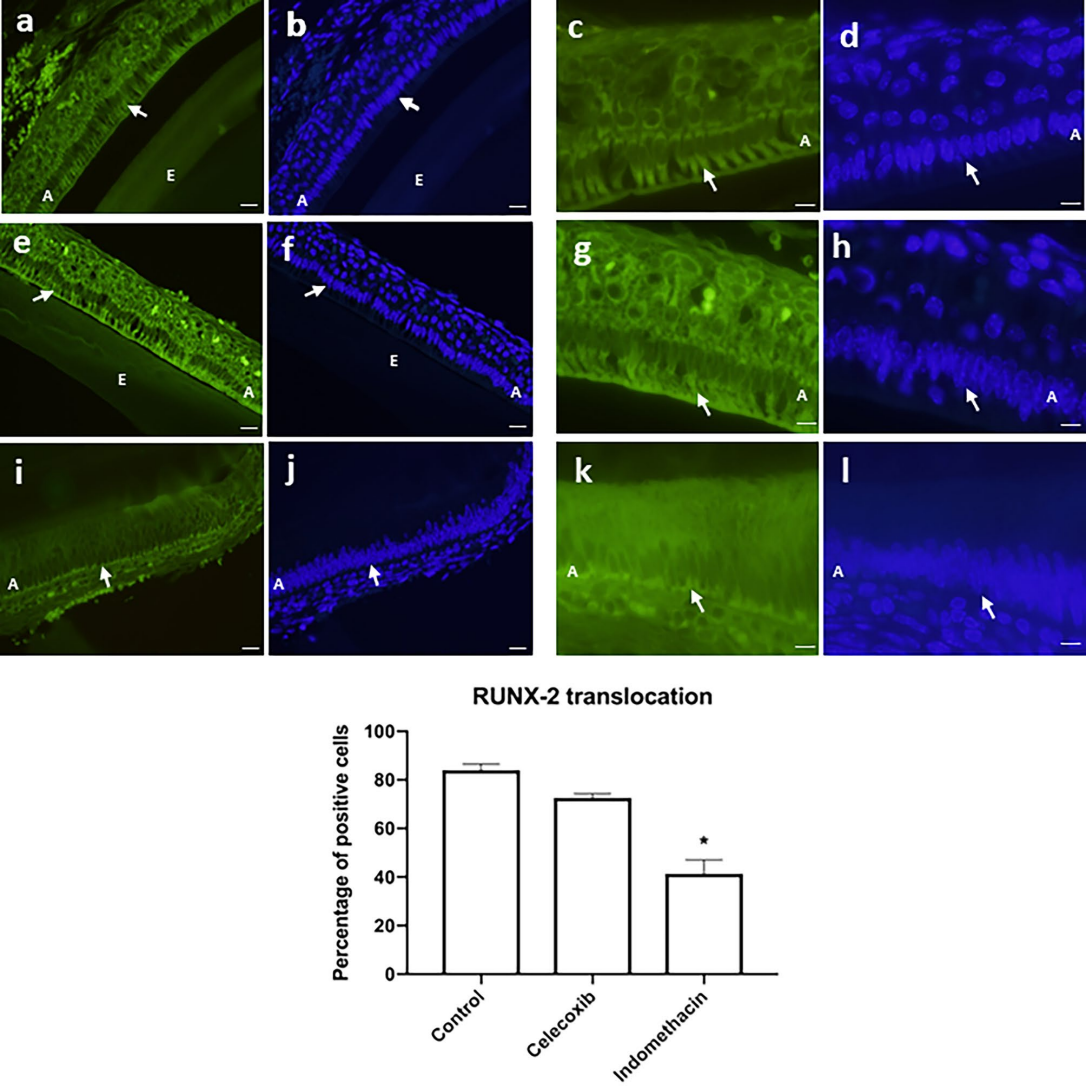
Fluorescence microscopic images showing Runx2 (cytoplasmic and translocated to the nucleus) in the control group (n = 20) with an intense perinuclear staining (40×*/*scale = 20 µm) (**a** and **b**), (100×*/*scale = 10 µm) (**c** and **d**). Celecoxib treatment (n = 20) was similar to the control group (n = 20) (40×*/*scale = 20 µm) (**e** and **f**), (100×*/*scale = 10 µm) (**g** and **h**). Indomethacin treated impaired nuclear translocation of Runx2 which was concentrated close to the basal layer (40×*/*scale = 20 µm) (**i** and **j**), (100×*/*scale = 10 µm) (**k** and **l**). A: ameloblasts, E: enamel layer.

Runx2 transcription factor presented a high expression in the ameloblast layer with a perinuclear immunostaining in the control group. In the groups treated with indomethacin or celecoxib, an inhibition of the transcription factor was observed (Fig. 6). Interestingly, we also found that Runx2 translocation to the nucleus reduced upon treatment with indomethacin was more robust than treatment with celecoxib (Fig. 7).

**Figure 8:**
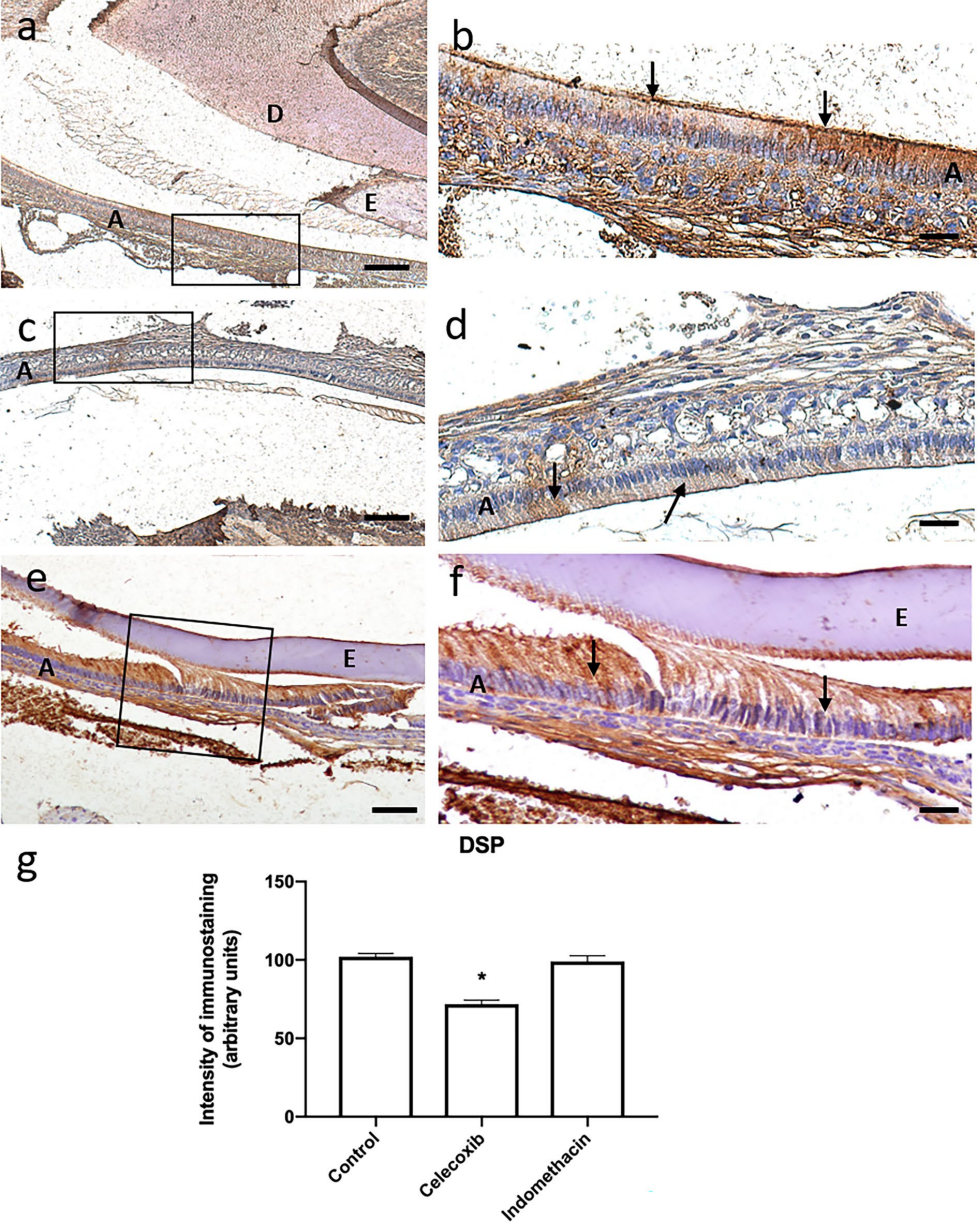
Control group (n = 20) shows intense DSP immunoreactivity (10×*/*scale = 100 µm) (**a**), (20×*/*scale = 50 µm) (**b**). Group treated with Celecoxib (n = 20) showed mild inhibition of DSP protein compared to control (10×*/*scale = 100 µm) (**c**), (20×*/*scale = 50 µm) (**d**), while Indomethacin-treated group (n = 20) showed more intense expression than celecoxib-treated group (n = 20) (20×*/*scale = 50 µm) (**e**), (20×*/*scale = 50 µm) (**f**). Graphical representation of immunostaining quantification of DSP protein synthesis using image deconvolution- median and interquartile range (**g**). A: ameloblasts, E: enamel layer, D: dentin layer.

In the control group, DSP showed an intense expression throughout the ameloblastic layer. It was found that as the ameloblasts were less differentiated, there was greater expression, and when they were more mature, the DSP expression becomes less intense. In the group treated with celecoxib, DSP was expressed, but less intensely than in the control group. While in the indomethacin group, expression was similar to the control group (Fig. 8).

## Discussion

In this study it was found that NSAIDs, medication commonly prescribed for children, impaired the synthesis and nuclear translocation of proteins and enzymes important for enamel biomineralization. Those findings shed light on the possibility that those drugs might be involved in the etiology of enamel defects. DEDs occur due to alterations during the period of secretion of the dental matrix in amelogenesis, and its severity will vary according to the period in which there is any disturbance^3,4^.

MMP-20 is an enzyme present in the extracellular matrix of enamel, being responsible for the degradation of the organic matrix, providing space for the growth of hydroxyapatite crystals and deposition of mineral content^10,22^. A low expression of MMP-20 in the control group was found. While the groups treated with indomethacin or celecoxib, a selective inhibitor of COX-2^23^, a high expression of MMP-20 was observed. Based on this result, it is hypothesized that MMP-20 deficiency alone might be not responsible for changes in the mineralization quality and cause of enamel defects.

Animals with a mutation on MMP-20 gene have been report to have a greater amount of organic content inside the hydroxyapatite crystals when compared to the group of animals that did not have the MMP-20 mutation^24^. From this, it was concluded that the absence of MMP-20 in the mineralization process can lead to the accumulation of amelogenin in hydroxyapatite crystals. The presence of these proteins inside the crystals hinders their expansion process, and they end up getting smaller. In another study^22^, the differences in enamel characteristics of animals with normal expression of MMP-20 in the maturation period and those without MMP-20 were investigated. It was reported that in the group with no enzyme, enamel had a lower hardness, a lower mineral amount and a higher amount of water and protein compared to normal enamel. Accumulation of MMP-20, on the other hand, in pre-ameloblasts has been reported to be responsible for causing enamel defects^25^.

Runx2 transcription factor is responsible for mediating bone formation. It participates in the differentiation of osteoblasts and in the osteoclastogenesis process around the dental follicle and periodontal ligament^26^. In an attempt to identify the functions that the transcription factor played in the amelogenesis process, an experiment was carried out with animals in which it was observed that the absence of Runx2 can cause damage such as delay in tooth mineralization and in the expansion of enamel crystals. A delay in the removal of proteins from the enamel extracellular matrix, a decrease in the KLK4 enzyme, which is responsible for protein degradation, and the accumulation of proteins in the matrix during the period of maturation have been reported^27^.

The present results show that the control group showed high expression of Runx2, different from the group treated with indomethacin and celecoxib, in which there was an inhibition of the transcription factor. Nonetheless, solely indomethacin prevented Runx2 translocation to the nucleus. Mutation in Runx2 cause limitations in enamel formation and bone formation^28^. It was observed that the mutation in the transcription factor caused alterations in the cell cycle of the dental pulp cells, decreased the cell proliferation rate and its calcification capacity^29^.

DSP is an important protein for dentin formation, and the deficiency of this protein is related to the occurrence of type III *dentinogenesis imperfecta*^13^. Interestingly DSP has been identified in the pre-ameloblast layer as a signaling molecule^30^, although its role in biomineralization of enamel is widely unknown. Recently, using a mutant DSPP mice, it was found that if DSPP remains retained in the endoplasmic reticulum within the presecretory ameloblasts and causes ER stress^25^. Because it has been shown that ER stress and its associated unfolded protein response (UPR) is involved in amelogenesis imperfect caused by mutations in the genes that encode enamel matrix proteins^31–33^. Liang et al.^25^ postulated that the enamel defects observed in DSPP deficient mice may be associated with ER stress and unfolded protein response. The present work sought to explore the presence of DSP on ameloblasts following NSAIDs treatment and found that it is constitutively synthesized in the layer of ameloblasts, contributing to similar evidence mentioned above. In addition to its presence, it was also possible to observe a greater amount in the region of ameloblasts that were in less differentiated stages, thus evidencing the participation of DSP in the earlier stages of enamel matrix secretion. When compared with the groups treated with NSAIDs, it was noticed that there was an inhibition, which was even more evident in the group treated with celecoxib. Despite still not knowing the mechanisms of action of DSP and its functions in amelogenesis, the use of medications can lead to its inhibition and, therefore, potentially damage to the process, causing possible defects in the enamel. Notwithstanding, the accumulation of DSPP in pre-ameloblast would eventually lead to the occurrence of disturbances in the mineralization process such as delay and malformation of dental enamel^14^. DSPP is an acidic protein, and its accumulation in the endoplasmic reticulum would cause stress. This condition of pathological stress on the endoplasmic reticulum would activate a phenomenon called unfolded protein response, and thus there would be a mutation of the protein, causing enamel defects.

COX-2 is considered pathological enzyme, since its presence has always been associated with inflammatory processes, but one study^15^ and the present study have identified the presence of COX-2 in physiological tooth enamel formation. COX-2 was expressed in the ameloblastic layer, with a higher expression in the less differentiated ameloblasts, which would be in the initial stage of mineralization. Therefore, because COX-2 appear to be physiological in enamel formation, this study sought to investigate whether medications commonly used in childhood such as NSAIDs would be involved in DEDs. It has been reported that there is a relationship between inhibition of COX-2 and a quantitative reduction in tooth enamel minerals, calcium and phosphorus, caused by the use of ibuprofen. Also, according to the authors, the inhibition of COX-2 activity would lead to a decrease in prostacyclin, which in turn would cause a decrease in blood flow in the dental organ, reducing the arrival of nutrients and entry of ions, and thus impairing the mineralization process^15^.

It is important to consider that animal models in dental experimental studies are widely used, due to the difficulty of access to developing teeth, which occur during intrauterine life inside the maxillary bones. Mice are an option as an animal model for enamel formation studies, even though they present morphological and numerical differences. It is known that the tooth formation process differs in some points, such as the renewal and growth of dental enamel due to the constant activity of ameloblasts along the incisors^6,34^. However, studies have shown the presence of important components such as the enzyme MMP-20 and the protein amelogenin in the composition of mice teeth^10,35^.

Therefore, the results described has showed that indomethacin can inhibit key proteins and enzymes in the dental mineralization process, and might be involved in DED etiology. DSP was also identified in the ameloblast layer, thus being a protein that plays a fundamental role in dentinogenesis, and its role in amelogenesis should be further investigated. It is important to emphasize the importance of carrying out more studies to identify the mechanisms of action that NSAIDs can actually exert to cause DDE if used in the period of tooth formation and mineralization.

## Methods

### Indomethacin and celecoxib treatment

Sixty C57BL/6 6-week-old male mice (*Mus musculus*) weighting 20–22 g were acquired from the Central Animal Facility at University of São Paulo in Ribeirão Preto. All animals were kept in Animal Facility at the School of Dentistry of Ribeirão Preto-USP, housed in polypropylene cages with stainless steel lids, 15 × 20 cm (3 animals per cage), lined with autoclaved in a light temperature (22 ± 10%) and relative humidity (55 ± 10%), in a light, on a 12:12 h light–dark cycle, with a standard laboratory diet, free access to autoclaved water. The experiment had the approval by the Ethics Committee on Animal Use (CEUA), from the Ribeirão Preto Campus, University of São Paulo (USP) (#13.1.266.53.6). The animals were treated according to Brazilian Guideline for the Care and Use of animals in Teaching or Scientific Research Activities regulated by the national Council for the Control of Animal experimentation (Law 11.794/2008). Animal studies were conducted in compliance with ARRIVE guidelines^36^.

The animals were assigned into three groups: celecoxib (n = 20) or indomethacin (n = 20) treatment for a period of 28 days or received no medication (control group; n = 20). The non-selective COX-1 and COX-2 inhibitor Indomethacin (C_19_H_16_ClNO_4_; Cayman Chemical, Ann Arbor, MI, USA) solution was prepared in saline with 5% NaHCO_3_ and was given i.p. (5 mg/kg), daily throughout the experimental period. Selective COX-2 inhibitor Celecoxib (C_17_H_14_F_3_N_3_O_2_S; Pfizer Inc., La Jolla, CA, USA) was prepared in ethanol and saline, being provided by gavage (15 mg/kg), daily throughout the experimental period. Healthy incisor teeth from animals that did not receive medication were used as control. The animals were anesthetized, then euthanized in the CO_2_ chamber, and tissues containing bone and tooth were collected for further analysis.

### Scanning electron microscopy–energy dispersive X-ray analysis

After the extraction of the lower incisors, the samples were dried, and then affixed to scanning electron microscopy (SEM) stubs, sputter-coated with carbon and examined with a JEOL-JSM-6610LV operating at 20 kV and 15–20 mm working distance. Quantitative element analysis was carried out with an Oxford Instruments INCA 300 EDX System (Abingdon, Oxfordshire, UK). The element content was calculated as the relative weight percentage of the total element content (100%). The count was conducted on the incisal and cervical areas of the lower incisors. The elements quantified were calcium (Ca), phosphorus (P), oxygen (O) and carbon (C). Statistical analyses of data were carried out using one-way ANOVA followed by Turkey test (α = 0.05).

### Knoop microhardness test

Lower incisors were dried and the test was performed with a load of 10 gf for 5 s in a microhardness testing machine (Shimadzu–HMV-2, Kyoto, Japan) equipped with a Knoop diamond tip. The indentations were performed in three regions: the first was made in the tip of the teeth, the second one in the middle area of the enamel, and the third next to cervical region. Statistical analyses of data were carried out using one-way ANOVA followed by Turkey test (α = 0.05).

### Micro-computed tomography

A high-resolution, desktop µCT system (Phoenix V tome xS240, GE, Boston, USA) was used to scan the samples. The samples were cleaned and dehydrated, and put in a plastic microtube. Scanning parameters were 70 kV and 200 µA, 0.1 mm Al/Cu filter and the voxel size was 5.4 μm. The projections were acquired over a full circle of rotation steps at 0.4° angle intervals, and each projection was composed of the average of 3 transmission images. The average time of scanning was around 2 h. The data from the tomography projection scans were reconstructed using the 3D Slicer Software^37^, and then analyzed using ImageJ (Wayne Rasband, National Institutes of Health, USA) software. Hydroxyapatite Ca_5_(PO_4_)_3_(OH) was used for standardization and calibration of measurements. The mean gray-value of the mineral grains were set to as the electron densities of 3.17 g/cm^3^. The volume, thickness and density of enamel was measured from the CT-scans central section of the incisors at the point just in front of the margin of the lower jaw bone.

### Histological processing

The jaws were dissected and removed with surgical scissors. The blocks containing incisor teeth and bone were fixed in 10% buffered formalin for 24 h at room temperature and demineralized in 10% EDTA (Merck S.A. Chemical Industries, Rio de Janeiro, RJ) for approximately 21 days. After demineralization, the samples were submitted to routine histological processing, washed in running water for 24 h, dehydrated in increasing concentrations of alcohol, diaphanized in xylol, and embedded in paraffin. The blocks were sectioned transversally, in an axial direction, to obtain cuts with a thickness of 5 µm. Sections were then stained by hematoxylin and eosin (HE) for histological evaluation.

### Immunohistochemistry

Slides were deparaffinized, hydrated in a decreasing ethanol series, and kept in phosphate-buffered saline (PBS). Next, tissue sections were microwaved (7 × 12 s at 2-min intervals) with sodium citrate buffer (pH = 6.0) for antigen retrieval. After temperature stabilization, the slides were washed with PBS (3×) for 5 min, and endogenous peroxidase activity was blocked with 3% hydrogen peroxide for 40 min. Slides were further washed with PBS (3×) for 5 min and non-specific binding sites were blocked with 5% bovine serum albumin (Sigma-Aldrich) for 60 min. The tissues were then incubated with primary antibody for COX-2 (sc-1746, Santa Cruz Biotechnology, Santa Cruz, USA), DSP H-300 (sc-33587, Santa Cruz Biotechnology), MMP-20 (sc-26926, Santa Cruz Biotechnology) and Runx2 (ab-23981, Abcam, USA) at 4 °C overnight. Next, slides were washed and incubated with mouse anti-goat (sc-2491, Santa Cruz Biotechnology) and mouse anti-rabbit (sc-2489, Santa Cruz Biotechnology) biotinylated secondary antibody for 1 h, washed in PBS, and incubated with streptavidin conjugated with horseradish peroxidase (HRP) for 20 min. 3,3′-diaminobenzidine (DAB, Sigma-Aldrich) was used as the enzyme substrate for 5 min; the slides were washed with PBS, counterstained with hematoxylin for 15 s, washed with distilled water, dehydrated in increasing ethanol concentrations, and mounted in Entellan^®^ (Merck, Darmstadt, Germany). Negative control slides, in which the primary antibody was omitted, were used to test the specificity of immunostaining. For quantification of intensity of the immunostaining in the ameloblast layer, the Software Image J (National Institutes of Health, Bethesda, MD, USA) and the image deconvolution plugin (Color Deconvolution) were used. DAB vector was applied and then the selected channel and threshold were manually adjusted, at 10× magnification^38^. Analysis was carried out by blinded examiners to the sample group assignment at the cervical third of the root, using in 1 section per slide, 1 tooth per animal, from 10 different mice. All immunohistochemistry staining for the same antibody was done in the same batch, with a rigid control of time and temperature that we described in the manuscript. Data obtained were analyzed using one-way ANOVA followed by Turkey test (α = 0.05).

### In situ zymography

Five μm-thick tissue sections were immersed in sodium borohydride (1 mg/ml) followed by incubation with a fluorescein isothiocyanate (FITC)-bound gelatin substrate (DQ™ Gelatin, Molecular Probes, Eugene, OR) dissolved in agarose (0.1 mg/ml) for 2 h at 37 °C in a humidified light-protected chamber. Nuclei were counterstained by adding 4′-6-Diamidino-2-phenylindole (DAPI; 0.5 μg/ml) to the incubation medium. Control slides were preincubated in 20 mM ethylene diamine tetraacetic acid (EDTA, Sigma, St Louis, MO) for 1 h, and then EDTA was added to the incubation medium. Quantification of gelatinolytic activity in the sections was assessed by counting the number of spots of fluorescence in the ameloblast layer (10× magnification) and expressed as number of spots of enzymatic activity *per* mm^2^.

### Indirect immunofluorescence

To investigate the translocation of runt-related transcription factor 2 and its role in the synthesis of biomineralization proteins and as an indicator of cell differentiation, indirect immunofluorescence assays were conducted.

The slides were prepared as described above. Next, the slides were washed with PBS (3×) for 5 min and 1 mg/mL sodium borohydride solution (3x) (Dinâmica Química Contemporânea Ltda., Diadema, SP, Brazil) for 15 min. Non-specific binding sites were blocked with 5% bovine serum albumin (Sigma-Aldrich) for 60 min. Immunolabeling was done using primary antibody for runt-related transcription factor 2 (ab-23981, rabbit polyclonal, Abcam) at 4 °C overnight. The following day, the slides were washed in PBS (3×) and incubated with secondary antibody (mouse anti-rabbit IgG conjugated with fluorescein) for 1 h in a dark chamber. The slides were further washed with PBS (3×) and the nuclei were stained with 4′,6-diamidino-2-phenylindole (DAPI) (0.5 µg/ml) (Santa Cruz Biotechnology Inc., Dallas, TX, USA) for 5 min. Slides were mounted with ProLong Gold Antifade (Molecular Probes Inc., Eugene, USA). The amount of positively stained cells was determined in the ameloblast layer and then the percentage of cells that presented Runx-2 translocated to the nucleus was calculated. First, the regions were photographed by fluorescence microscopy at 20× magnification using fluorescein isothiocyanate (FITC) and DAPI filters. Next, the images were analyzed using Image J software (U.S. National Institutes of Health, Bethesda, MD, USA) to determine the number of positively stained cells by and selecting the “*analyze particles*” tool: pixel size was set as the average size of previously measured cell nuclei. Total cell count and stained cell count were expressed as a percentage and staining was compared across groups using one-way ANOVA followed by Tukey’s test (α = 0.05).

## Data Availability

The data that support the findings of this study are available from the corresponding author, F.W.G.P.S, upon reasonable request.
